# A case report and reconsideration of pathogenesis: C5 palsy after corrective surgery for severe congenital cervicothoracic scoliosis

**DOI:** 10.3389/fsurg.2026.1767159

**Published:** 2026-04-22

**Authors:** Bo Zhou, Qihui Duan, Wenjin Li, Li Zhang, Tao Li, Zhi Zhao, Yingsong Wang

**Affiliations:** Department of Orthopedics, The Second Affiliated Hospital of Kunming Medical University, Kunming, China

**Keywords:** C5 palsy, case report, congenital scoliosis, hyperbaric oxygen therapy, postoperative complication

## Abstract

**Objective:**

This case report presents a rare case of C5 nerve root palsy following corrective surgery for severe congenital cervicothoracic scoliosis in a child, and explores its distinctive pathogenesis and treatment strategy.

**Methods:**

This study was approved by the Medical Ethics Committee of the Second Affiliated Hospital of Kunming Medical University (PJ-2021-100), and informed consent was obtained from the patient's guardian. The clinical data of a 9-year-old boy with congenital cervicothoracic scoliosis (Cobb angle of 99°) who underwent posterior corrective instrumentation and fusion were retrospectively analyzed. Typical C5 palsy developed on postoperative day 7. Imaging studies and the clinical course were used to identify the responsible mechanism.

**Results:**

Postoperatively, left deltoid strength decreased to grade II/III. CT excluded direct implant impingement, and no laminectomy had been performed, thus excluding the classic “posterior cord drift” hypothesis. Comparative imaging revealed a significant reduction in the vertical diameter of the C4–C5 foramen. We concluded that reduction maneuvers had transmitted excessive traction through the C5 pedicle screw, producing dynamic foraminal stenosis and nerve root stretching. Conservative management (with cervical traction and hyperbaric oxygen) was instituted, and complete neurological recovery was documented at 3 months.

**Conclusion:**

In cervical deformity surgeries performed without decompression, iatrogenic alteration of the foramina’s geometry, produced by corrective forces, is an important and frequently overlooked mechanism of postoperative C5 palsy. Surgeons should avoid overtightening a single screw during rod reduction; once the complication occurs, systematic conservative treatment usually yields a favorable outcome.

## 1 Introduction

C5 nerve root palsy is a well-documented complication of posterior cervical procedures and is classically ascribed to traction on the C5 nerve root after laminectomy-induced posterior cord drift. We recently observed a case of C5 palsy that did not fit this paradigm during the correction of rigid congenital thoracic-cervical scoliosis (Cobb angle of 99°) in a young child. The following three features distinguished this case: (1) severe, long-standing deformity in an immature spine; (2) instrumentation and fusion only—no laminectomy or direct cord manipulation; and (3) unchanged intraoperative neurophysiological signals. We postulate that acute foraminal narrowing produced by corrective maneuvers created a dynamic stenosis that transiently entrapped the C5 root. This report introduces a novel, non-laminectomy mechanism for postoperative C5 palsy and suggests preventive strategies during deformity correction.

## 2 Case report

### 2.1 Clinical presentation

A 9-year-old boy with normal intelligence and proportionate short stature (height: 120 cm; weight: 21 kg) was referred for a 4-year history of progressive cervicothoracic deformity and compensatory head tilt. There was no antecedent medical or surgical intervention, and neurological screening (motor strength, sensation, reflexes, and long-tract signs) was entirely normal upon admission. Physical examination revealed rigid right-sided cervical scoliosis with a prominent razorback ridge, an absence of tenderness over the spinous processes, incomplete segmentation of the right thumb, and an accessory auricle on the left. Preoperative pulmonary function tests indicated mild restrictive ventilatory dysfunction: Forced vital capacity (FVC) was 72% of predicted values, forced expiratory volume in one second (FEV1) was 70% of predicted values, and the FEV1/FVC ratio was 85%. This corresponded to reduced chest volume due to severe cervical-thoracic deformity.

**Figure 1:**
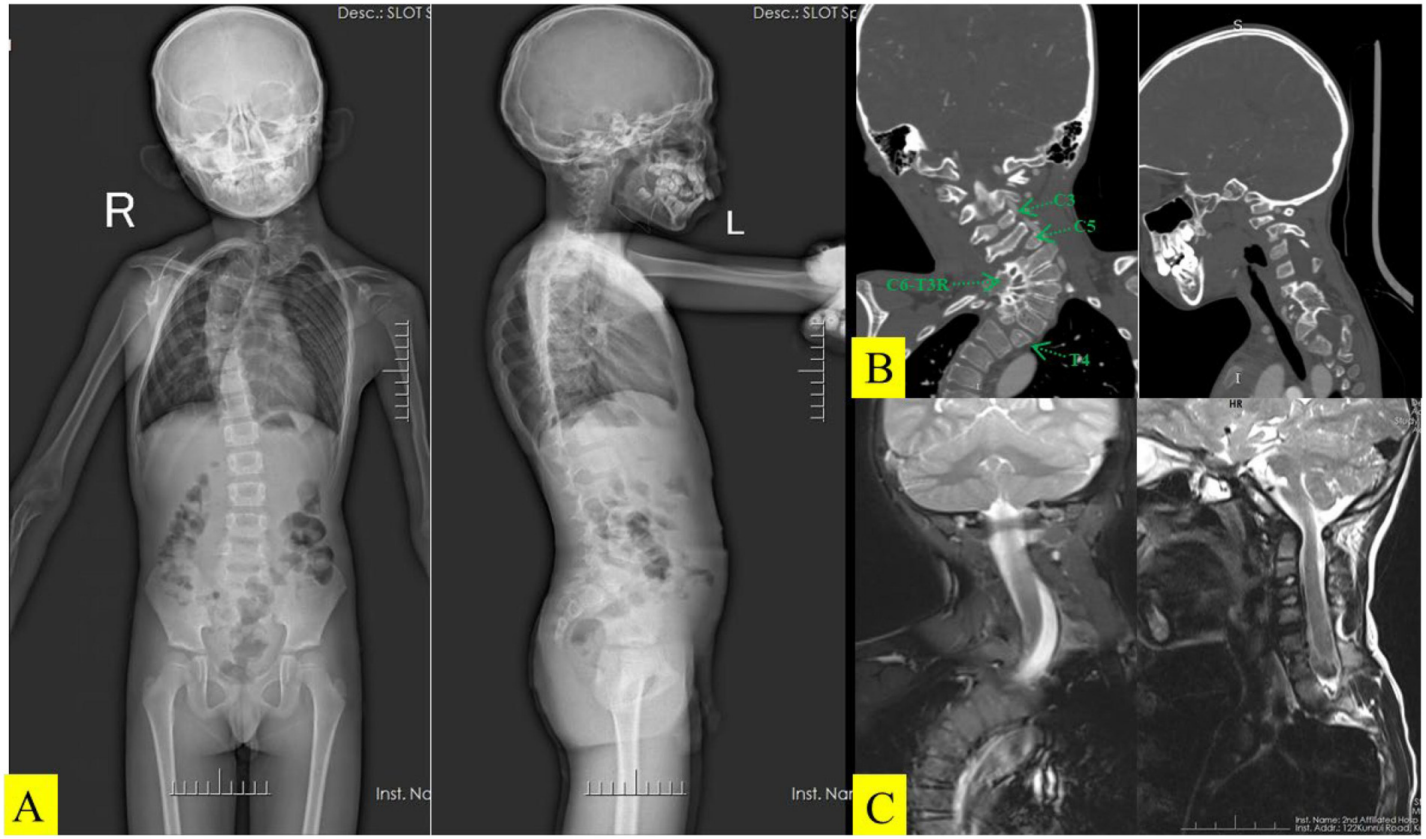
**(A)** Preoperative full-length anteroposterior and lateral spine X-ray images of the patient in the standing position. **(B)** Three-dimensional computed tomography imaging suggests that C3, C5, and T4 are butterfly vertebrae and that the area from C5 to T4 exhibits intervertebral fusion on the concave side. **(C)** Preoperative MRI of the cervical and thoracic spine suggests that there are no intraspinal malformations or degeneration of the spinal cord.

The patient presented with isolated congenital scoliosis. Standing full-length radiographs showed a cervicothoracic curve from C5 to T8 with Cobb angles of 99° in the cervical segment and 46° in the thoracic segment (Figure 1A), with no signs of neuromuscular disorders. Genetic testing and comprehensive screening did not reveal any syndromic abnormalities, confirming that this was a non-syndromic sporadic case. Preoperative neurological function was assessed using the modified Japanese Orthopaedic Association (mJOA) scoring system (1), which yielded a score of 17/18 (indicating a mild spinal cord injury). Although there was mild sensory loss in the C5 dermatome, there were no motor deficits or gait abnormalities. Three-dimensional CT demonstrated butterfly vertebrae at C3, C5, and T4, together with concave-side auto fusion from C5 to T4 (Figure 1B). An MRI of the cervical and thoracic cord excluded intraspinal anomalies (Figure 1C).

### 2.2 Preoperative halo-gravity traction

**Figure 2:**
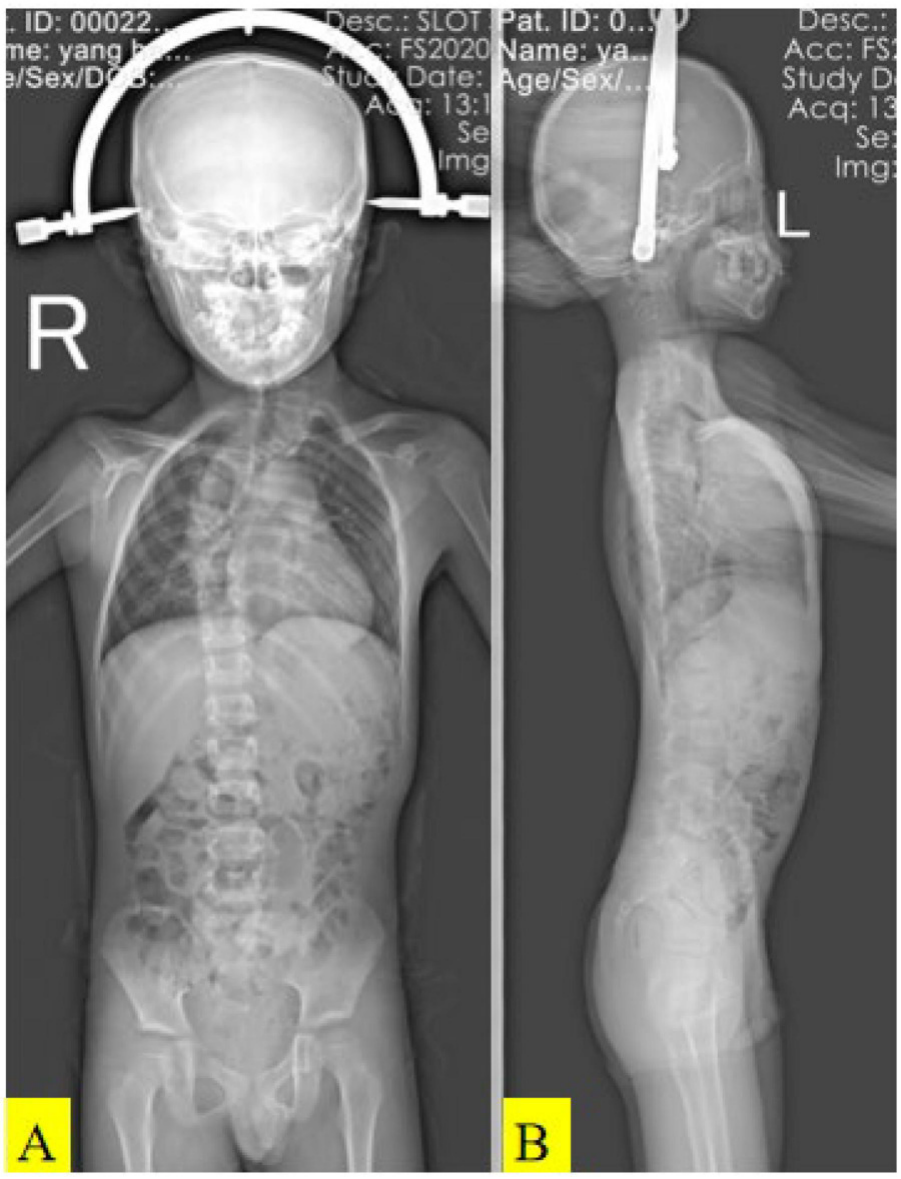
Anteroposterior and lateral X-ray images of the patient's spine in the traction position **(A, B)**.

Halo gravity traction was commenced immediately upon admission (Figure 2). Weight was increased incrementally from 5 to 9 kg over 3 weeks, with weekly radiographs to monitor curve flexibility. No neurological deterioration or new motor deficits were observed during traction.

### 2.3 Operative procedure

Because of severe local dysplasia and hyperplastic pedicles at the cervicothoracic junction, screw purchase was expected to be marginal; correction, therefore, relied on continued traction plus limited posterior instrumentation. After induction of anesthesia, 3 kg of cranial traction was maintained, and multimodal intraoperative neurophysiological monitoring [motor evoked potentials (MEPs) + somatosensory evoked potentials (SSEPs)] was instituted. A posterior midline approach from C3 to T10 was performed. Fifteen pedicle screws (C3–T10) were inserted under fluoroscopic guidance. A domino connector was used on the right side to link two rods and allow gradual, manually controlled derotation while continuous MEP signals remained stable. The facet capsules and cortical laminae were decorticated with a high-speed burr, and the fusion bed was packed with cancellous allograft. The wake-up test was normal before closure.

### 2.4 Postoperative course

**Figure 3:**
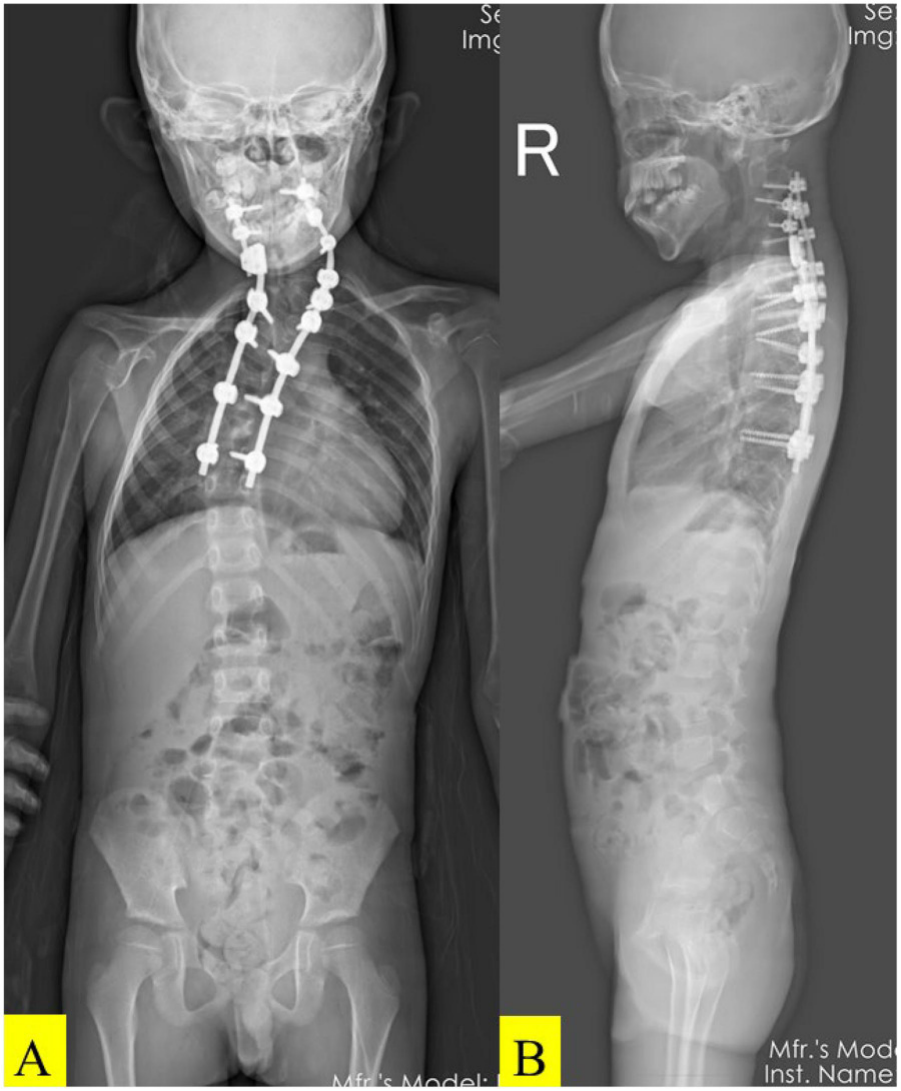
Postoperative standing anteroposterior and lateral X-ray images of the full-length spine. The Cobb angle of the cervical segment was 46°, and that of the thoracic segment was 40° **(A, B)**.

The patient awakened neurologically intact. Immediate postoperative radiographs showed correction of the cervical Cobb angle to 46°and the thoracic Cobb angle to 40° (Figure 3). A rigid cervical collar was applied.

**Figure 4:**
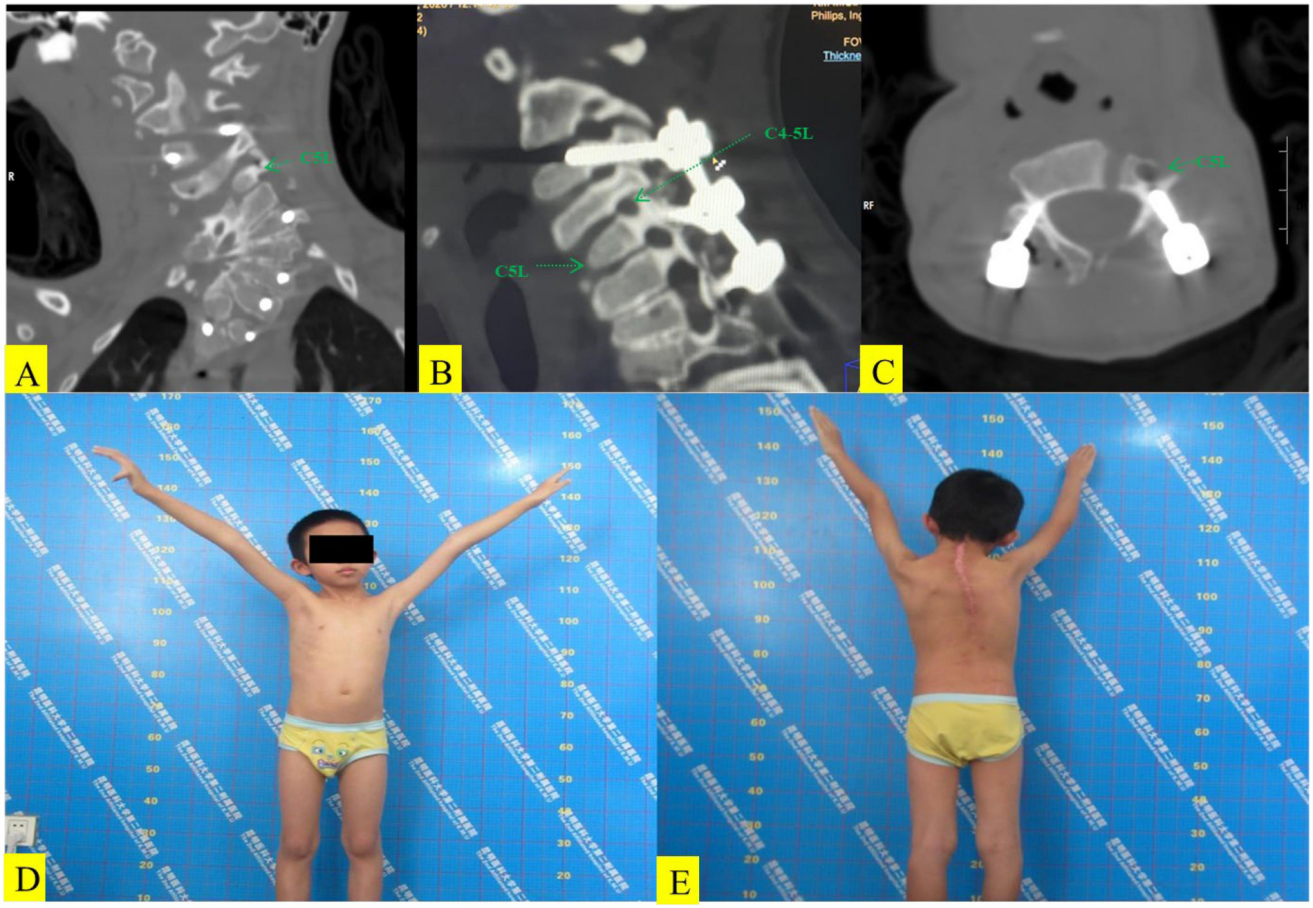
Postoperative CT image of the cervical spine reveals that the nail tract for internal fixation is satisfactory **(A–C)**. At the 3-month follow-up, the myodynamia of the right deltoid muscle is normal **(D,E)**.

On the third day after surgery, induced spirometry training and respiratory physiotherapy were initiated. Three months after surgery, pulmonary function tests showed that FVC increased to 82% of the predicted value, and FEV1 increased to 80% of the predicted value, indicating an improvement in chest wall mechanics after orthopedic surgery. This is consistent with literature reports indicating that orthopedic treatment for congenital scoliosis can improve children's respiratory function (2). On postoperative day 7, the child developed bilateral periscapular pain [visual analog scale (VAS) score: 5–6] and an inability to abduct or forward-flex his left shoulder. Sensation remained intact; manual muscle testing revealed deltoid 2/5, triceps 3/5, and normal wrist/hand function—findings consistent with an isolated left C5 nerve root palsy. CT confirmed satisfactory screw position without foraminal encroachment (Figures 4A–C). The left shoulder was placed in an abduction sling, and mannitol and analgesics were administered. Because symptoms persisted, gentle halo traction (2 kg) was re-instituted on day 14. After 3 weeks, his pain decreased to a VAS score of 1–2; his deltoid strength improved to 3+/5, and his triceps strength to 4+/5. Traction was discontinued, and a 4-week course of hyperbaric oxygen therapy was completed while the collar was maintained. At the 3-month follow-up, the patient exhibited full, painless abduction of his left shoulder (Figures 4D,E), his deltoid and triceps strength had returned to 5/5, light-touch and pin-prick sensation were normal, and his mJOA score improved to 18/18, indicating complete resolution of the C5 palsy.

## 3 Discussion

C5 palsy (C5P) is the most common iatrogenic neurological complication after posterior cervical surgery. Clinical symptoms comprise new or worsened deltoid weakness. They are often accompanied by biceps involvement, radicular pain, or C5-dermatomal hypoesthesia developing after cervical decompression and are typically unilateral (3–6). In addition, deltoid weakness induces a loss of active shoulder abduction, which can severely compromise shoulder girdle function and quality of life.

Congenital scoliosis (CS) is intrinsically high-risk, including rigid, multi-planar deformity, poorly mineralized pediatric bone, and prolonged operative time, which combine to magnify neurological morbidity (7). Duan et al. reviewed 481 patients with CS and reported an overall neurological complication rate of 6.96%; when the preoperative Cobb angle exceeded 100°, the incidence rose to 30.23% (8). In a multicenter cohort of 1,373 scoliosis surgeries, Qiu et al. documented a 2.89% incidence in 381 patients with CS—significantly higher than the 1.89% observed in adolescent idiopathic scoliosis (9). The presence of a split-cord malformation further increases the risk to 5.1% (10). Our report presents a patient with a cervicothoracic Cobb angle of approximately 100°, multiple butterfly vertebrae, and concave-side auto fusion who clearly belongs to this ultrahigh-risk subgroup. Although preoperative halo gravity traction was well-tolerated and MRI excluded intraspinal anomalies, the magnitude of the deformity alone mandated maximal vigilance by the physician. It is worth noting that the surgical strategy for this patient only consisted of “traction-based pre-correction+*in situ* fixation”; no laminectomy, foraminotomy, or osteotomy was performed. This background differs fundamentally from the “decompression-related” C5P cases previously reported, and prompted us to explore an alternative pathogenesis.

The prevailing “posterior cord drift–nerve root tethering” hypothesis holds that wide decompression allows the spinal cord to migrate dorsally; the short, fixed C5 root is then stretched over the uncinate process, compromising its microvasculature and function (11, 12). In the present case, no decompressive procedure was undertaken, and the cord remained in its native position, rendering the classical explanation untenable.

**Figure 5:**
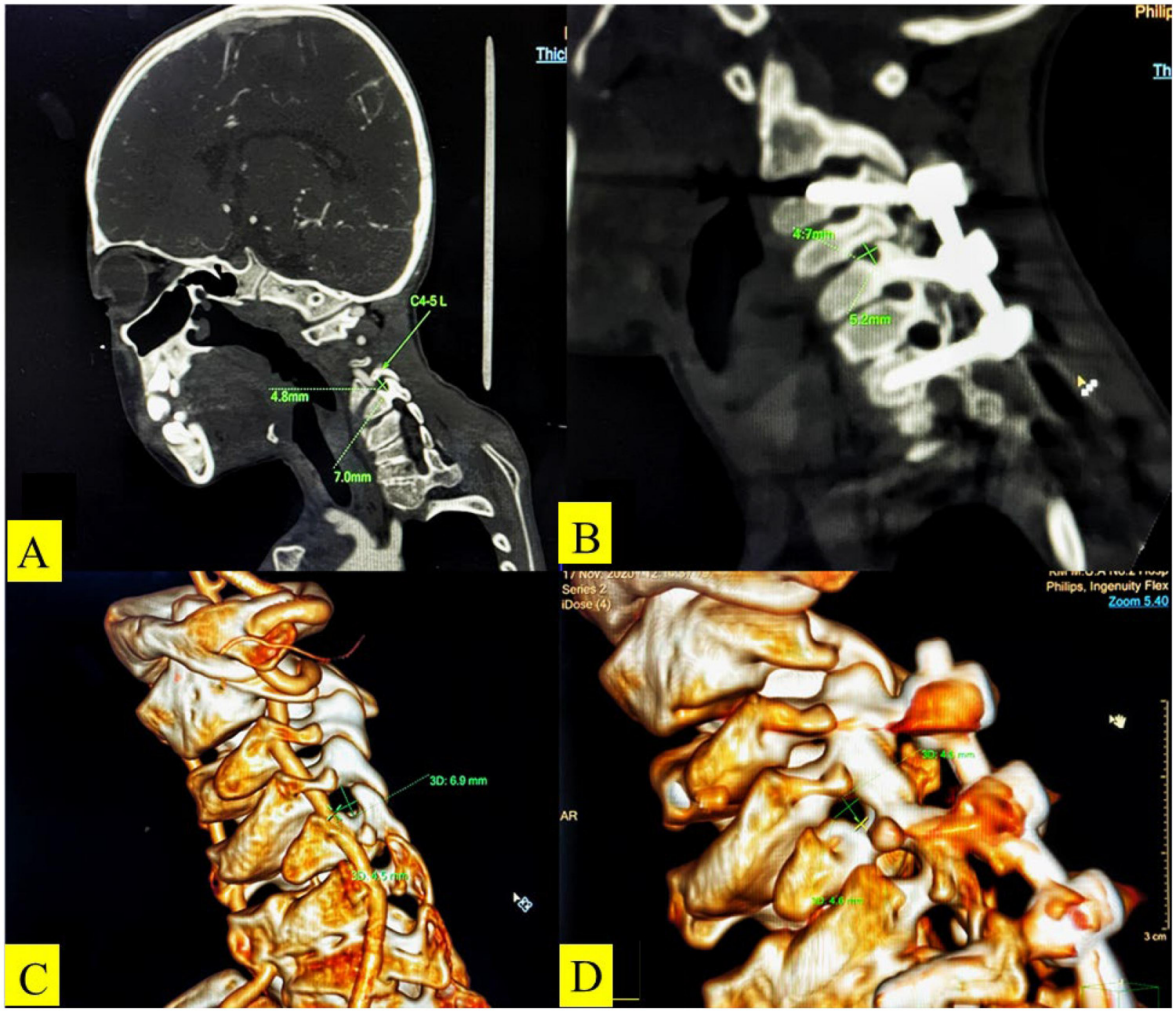
The vertical diameter of the right intervertebral foramen of C4-5 is 7.0 mm preoperatively **(A)** and 5.2 mm postoperatively **(B)** on the coronal CT images. Three-dimensional CT images reveal that the vertical diameter of the right intervertebral foramen of C4–5 is 6.9 mm preoperatively **(C)** and 4.6 mm postoperatively **(D)**.

On postoperative day 7, the child developed typical left-sided C5 signs. Plain films and thin-cut CT confirmed that no screw had breached the spinal canal or intervertebral foramen, excluding direct, implant-related injury. Compared with preoperative CT, postoperative CT demonstrated that the gentle elevation (approximately 1.2 mm) of the left C5 pedicle screw to facilitate rod insertion induced a subtle lateral shift of C5 relative to C6 and reduced the vertical diameter of the left C5/6 foramen by 15% (Figure 5). This created a “dynamic exit stenosis” while simultaneously imparting longitudinal traction to the C5 root at the fixed uncinate process. Axonal microdamage and venous congestion presumably followed, culminating in clinical palsy. This “in situ fixation-dynamic foraminal stenosis” mechanism resembles the “vertebral translation-root impingement” described after osteotomies. However, to our knowledge, this has not previously been reported after only *in situ* instrumentation. Furthermore, chronic preoperative stretching (Cobb angle of approximately 100°) likely placed the C5 root microvasculature in a borderline ischemic state; even minor additional tension could have triggered a local reperfusion insult, synergistically amplifying edema and conduction block.

Modern multimodal intraoperative neuro-monitoring (IONM) has markedly reduced the incidence of global spinal cord injury, but its sensitivity to isolated, reversible root ischemia is limited. Zhang et al. observed that when MEP/SSEP signals are attenuated but not lost, the probability of a postoperative neurological deficit is low and recovery is favorable (13). In our case, IONM remained stable throughout, but C5P still developed, reinforcing the need for continued vigilance even when monitoring is uneventful.

C5P typically manifests 3–7 days after surgery. Published series indicate that >90% of patients recover at least partially and >50% regain full strength, usually within 6 months. Deltoid manual muscle testing (MMT) at onset is strongly predictive; previous studies have shown that an MMT ≥3/5 almost invariably normalizes, whereas an MMT ≤2/5 may portend incomplete recovery (3, 11). Our patient presented with a deltoid MMT of 2/5 (definitely an incomplete lesion). Early halo traction (2 kg) combined with hyperbaric oxygen was instituted, and full strength returned within 3 months, obviating the need for revision surgery. Although low-weight traction of 2 kg after surgery was successfully used to treat C5 paralysis without complications, this procedure theoretically carries risks, such as screw displacement, rod fracture, or loss of correction, especially in children with poor bone quality. In this case, weekly X-rays showed stable internal fixation and good maintenance of correction, indicating that the cautious use of low-weight traction is safe. Radiographs at 6 months confirmed solid fusion without loss of correction. Hyperbaric oxygen therapy has gradually gained attention in recent years as an adjunctive treatment for nerve injuries. Studies by Brenna et al. and Huang et al. have also confirmed the positive effects of hyperbaric oxygen on neural function recovery (14, 15). The treatment experience in this case suggests that combining hyperbaric oxygen therapy with conventional conservative treatments may have a synergistic effect on postoperative C5 paralysis. However, further research is needed to verify its exact therapeutic efficacy.

In summary, we describe a novel etiology of C5P, namely, “in situ fixation-induced dynamic foraminal stenosis,” that expands the pathogenic spectrum beyond classic posterior cord drifts. Recognition of this mechanism should refine surgical techniques and enhance surveillance strategies for complex congenital cervicothoracic scoliosis. Furthermore, this report provides some key lessons for physicians. First, in severe, rigid pediatric scoliosis, the main correction should be achieved by preoperative traction; forceful single-screw reduction during rod seating must be avoided to prevent dynamic foraminal stenosis. Second, normal IONM does not exclude postoperative isolated root injury; awareness of the dynamic foraminal mechanism is essential. Finally, early strength documentation, protected traction, and adjuvant hyperbaric oxygen constitute effective non-operative management of C5P and frequently avert reoperation.

This study has the following limitations. First, the proposed mechanism of “dynamic intervertebral foramen stenosis” is mainly based on imaging and clinical course observations. Second, the impact of postoperative edema or inflammatory reactions on nerve root ischemia remains speculative. Future studies with large sample sizes, combined with advanced technologies such as diffusion tensor imaging, are needed to verify this pathogenic mechanism.

## Data Availability

The original contributions presented in the study are included in the article/Supplementary Material, further inquiries can be directed to the corresponding authors.
